# The utility of liquid biopsy-based methylation biomarkers for colorectal cancer detection

**DOI:** 10.3389/fonc.2024.1351514

**Published:** 2024-03-26

**Authors:** Holli A. Loomans-Kropp

**Affiliations:** ^1^Cancer Control Program, Arthur G. James Cancer Hospital and Richard J. Solove Research Institute, The Ohio State University, Columbus, OH, United States; ^2^Division of Cancer Prevention and Control, Department of Internal Medicine, College of Medicine, The Ohio State University, Columbus, OH, United States

**Keywords:** liquid biopsy, colorectal cancer, methylation, biomarkers, early detection, screening

## Abstract

Colorectal cancer (CRC) is one of the most prevalent cancers and the second leading cause of cancer-related deaths in the United States. It is also one of the few cancers with established screening guidelines, however these methods have significant patient burden (e.g., time, invasive). In recent years, the development of liquid biopsy-based screening methods for biomarker detection have emerged as alternatives to traditional screening. Methylation biomarkers are of particular interest, and these markers can be identified and measured on circulating tumor and cell-free DNA. This perspective summarizes the current state of CRC screening and the potential integration of DNA methylation markers into liquid biopsy-based techniques. Finally, I discuss limitations to these methods and strategies for improvement. The continued development and implementation of liquid biopsy-based cancer screening approaches may provide an acceptable alternative to individuals unwilling to be screened by traditional methods.

## Opportunities to improve colorectal cancer screening adherence: blood-based screening tests

Colorectal cancer (CRC) is one of the most prevalent cancers and the second leading cause of cancer-related deaths in the United States (1). CRC is one of only four cancers (breast, lung, cervical) that has established United States Preventive Services Task Force (USPSTF) screening recommendations. Though there are methods to screen for prostate and skin cancers, screening is currently not recommended for prostate cancer and evidence showing benefit for skin cancer screening is insufficient to provide USPSTF recommendations (2, 3). USPSTF recommends CRC screening for all adults aged 45 to 75 years by (1) colonoscopy or flexible sigmoidoscopy with fecal immunochemical test (FIT) every 10 years (2), computed tomography colonoscopy (CTC) or flexible sigmoidoscopy every five years (3), high-sensitivity guaiac fecal occult blood test (FOBT) or FIT annually, or stool DNA-FIT every 1 to 3 years (4). The decision to screen between ages 76 and 85 should be made through physician-patient shared decision-making. Among the various screening modalities, colonoscopy is used most often in the United States, though there has been a noted increase in the use of stool DNA-FIT (5). However, with the expansion of screening options and recent alterations to the screening recommendations (e.g., lowering the screening age to 45), preferences may change. A novel conjoint analysis study of individuals aged 40 and older found that, if given the option between five different CRC screening tests (annual FIT, stool DNA-FIT every 3 years, capsule endoscopy every 5 years, CTC every 5 years, or colonoscopy every 10 years), approximately one-third of respondents expressed preference for a stool DNA-FIT test every three years, and this preference did not differ by age groups (45-49 years versus ≥50 years) (6). However, a separate study showed that preference for a stool-based test was either less or no different than colonoscopy (7). Despite colonoscopy remaining the most used CRC screening option in the United States, providing additional, less cumbersome alternatives may improve screening rates (8).

Despite these data and an assortment of screening options, many eligible adults do not get screened. Data from the National Health Interview Survey showed that 59% of individuals 45 and older were up to date with CRC screening in 2021 and rates were low for those age 45-49 (9, 10). A potential means to improve low adherence may be the utilization of high sensitivity blood-based CRC screening tests. A recent study of screening eligible individuals found high preference for blood-based screening; after declining the option of colonoscopy, 93.5% of participants elected for a blood-based screening option (11). Similarly, a clinic-based study in Germany found that, among participants who refused colonoscopy, 97% opted for a non-invasive method and, of those individuals, 83% chose a blood-based screening test over a stool-based test (12). These and other data show that, if provided the option, a substantial number of screen eligible individuals would prefer a blood-based option over traditional CRC screening methods. Despite this preference, currently, in the United States, there is only one FDA-approved blood-based CRC screening test (Epi proColon®), approved for use if first-line screening methods are declined (13).

## The utility of methylated DNA detection

**Figure 1:**
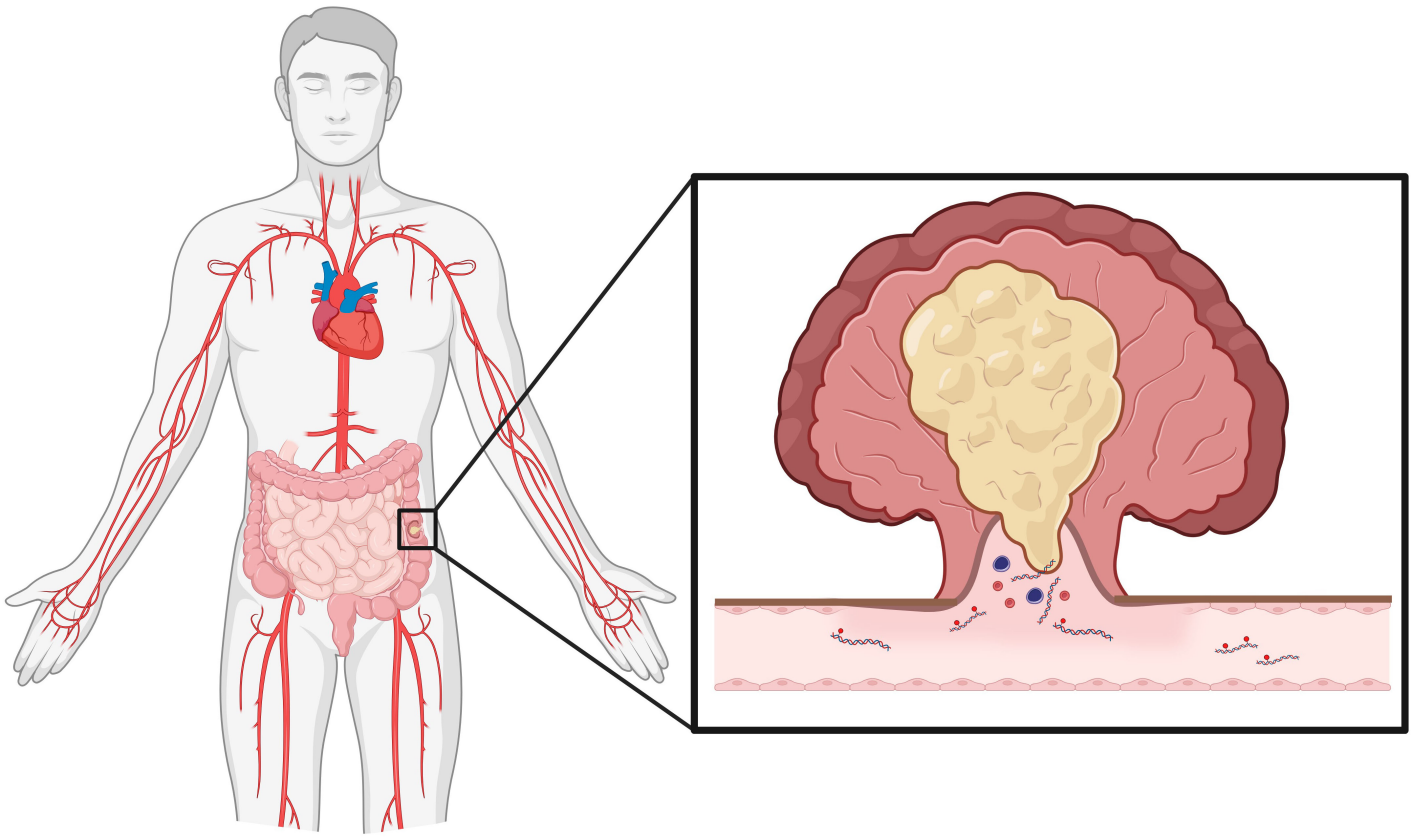
Colorectal cancer tumors shed cellular debris into circulation. Of interest for cancer detection, colorectal cancer cells release DNA fragments that contain methylation markers. As discussed in the perspective, methylation markers on these fragments may have substantial utility in minimally invasive cancer detection. (Created in Biorender).

The use of liquid biopsy, specifically blood-based methods, for cancer detection is an area of extensive research interest. Blood contains many types of measurable tumor-derived markers, such as circulating tumor cells, circulating tumor DNA (ctDNA) and cell-free DNA (cfDNA). Specifically, tumor-derived circulating cell fragments contain many distinct markers that may have utility in cancer detection, one such candidate marker being methylation (cell-free methylome) (**Figure 1**). Tumors cells exhibit genome-wide differential and abnormal methylation at specific CpG islands, often observed as hypermethylation of tumor suppressor genes and hypomethylation of oncogenes, commonly found in promoter regions and noncoding repetitive sequences (14, 15). An *in vitro* examination of fifteen CRC cell lines found differential methylation status among key tumor suppressor genes (*CDKN2A/p14^ARF^* and *CDKN2A/p16^INK4A^*), as well as several candidate genes, including *SCARA5* (16). Gene expression could be restored with 5-Aza-2’-deoxycitidine (5-Aza) treatment, which induces DNA demethylation. Moreover, treatment of five cell lines with 5-Aza induced upregulation of gene associated with tumor suppression (e.g., *CDKN1A, CDKN1C, NF1, SMAD3*), further demonstrating hypermethylation of tumor suppression pathways.

Unlike many other cancer types, CRC can be molecularly classified using tumor tissue, with methylation profiling being a key component of this characterization (17, 18). After several iterations of molecular subtyping, consensus subtyping established gold standard classifications of four CRC subtypes, all which incorporate methylation status (19). Briefly, consensus molecular subtype 1 (CMS1), which makes up 14% of diagnosed CRCs, exhibits global hypermethylation (CpG island methylator phenotype (CIMP)) with mutant *BRAF*, high microsatellite instability (MSI-H), and low somatic copy number alterations (SCNA); CMS2 is the canonical pathway, represents 37% of tumors, and shows a high number of SCNA, microsatellite stability, activated Wnt and Myc pathways, upregulated *EGFR*, and mutated *p53*; CMS3, the metabolic pathway and 13% of CRCs, exhibit a mixed phenotype - intermediate methylation profile and CIMP status, low MSI, moderately activated Wnt/Myc signaling, mutated *KRAS* and *PIKCA*, overexpressed IGBP3; and CMS4, a mesenchymal phenotype and 24% of CRCs, includes upregulation of genes relating to epithelial-to-mesenchymal transition, matrix remodeling, angiogenesis, and inflammatory-related signaling, and negative CIMP status (14, 20). Despite the differences among CRC molecular subtypes, all involve alterations in methylation profiles (21). Furthermore, these subtypes or profiles can inform treatment options. For example, MSI-H subtypes (CMS1) have been shown to respond better to PD-1/PDL-1 immunotherapy, due to increased immune cell infiltration, and TGFβ inhibitors have shown promise in the treatment of CMS4 tumors (20). However, there are limited therapeutic options specifically targeting methylation/CIMP, rather methylation markers may have better capability in CRC detection, as it is thought to occur early in the carcinogenic cascade (22).

## Candidate methylation markers for colorectal cancer detection

Many of the current applications used to ascertain methylation markers (e.g., CIMP status) require tumor tissue. However, as discussed previously, liquid biopsy-derived ctDNA and cfDNA fragments contain detectable methylation markers. Methylation has already, to an extent, been integrated into clinical practice; for example, Cologuard® (Exact Sciences), an approved multi-target stool DNA test, includes two methylation markers (*BMP3, NDRG4*), along with seven distinct *KRAS* mutations and hemoglobin, while Epi procolon®, an FDA-approved blood-based CRC detection test, relies solely on detection of methylated *SEPT9* (m*SEPT9*) and uses dichotomous absence/presence for outcome determination (18). In a clinical trial, Cologuard® showed 92.3% (95% CI: 83.0%, 97.5%) and 93.3% (95% CI: 83.8%, 98.2%) sensitivity for detecting any stage cancer and stages I-III, respectively, but only 42.4% (95% CI: 38.9%, 46.0%) sensitivity for detecting advanced precancerous lesions (23). A recent meta-analysis of studies utilizing m*SEPT9* for CRC demonstrated a pooled sensitivity and specificity of 0.69 (95% CI: 0.62, 0.75) and 0.92 (95% CI: 0.89, 0.95). Our group recently showed high sensitivity (90.8%; 95%CI: 84.7%, 96.9%), specificity (88.9%; 95%CI: 77.0%, 100.0%) and positive predictive value (96.3%; 95%CI: 92.3%, 100.0%) for m*SEPT9* in discerning early-onset CRC cases (<50 years old) from healthy aged matched and older (>50 years old) controls and that m*SEPT9* was an independent predictor of prognosis (24). Subsequent studies have combined m*SEPT9* with other markers to improve overall CRC detection (25). Due to substantial differences in profiles between healthy and cancerous cells, expanding detection panels to include multiple methylation markers found explicitly in CRC may improve clinical capability and usefulness.

Since the development and implementation of these tests, additional methylation markers have been suggested to improve liquid biopsy-based CRC detection, with brief summaries of four candidate biomarkers, included below.

### MutL Homolog 1

*MLH1* is often discussed in the context of Lynch syndrome, a hereditary predisposition to CRC and other cancers, as 35-40% of individuals with Lynch syndrome have a pathogenic mutation in *MLH1* (26). MLH1 is a component of the MutL complex, which critical for proper DNA mismatch repair (MMR), and loss of MLH1 results in MMR complex instability and defective MMR. However, MLH1 loss has also been observed in 4-20% of sporadic CRC and a small percentage of individuals with Lynch syndrome, a result of *MLH1* promoter hypermethylation (m*MLH1*) (27–29). Despite the relative frequency of m*MLH1* in CRC, a recent case-control study showed no difference in *MLH1* methylation status between CRC and healthy controls (30). Though m*MLH1* detection alone may have limited utility in average risk populations, *MLH1* can be detect in low abundance using digital drop PCR (ddPCR). Wang et al. demonstrated detection of m*MLH1* in plasma-derived DNA down to 0.096 ng using ddPCR (31). Majority of the research involving m*MLH1* detection is in Lynch syndrome, despite the high frequency of *MLH1* Hypermethylation in sporadic CRC, highlighting a gap in the field and opportunity for further exploration.

### Syndecan-2

*SDC2*, also called fibroglycan, is a heparan sulfate glycosaminoglycan-containing cell surface protein, which functions in cell adhesion and signaling. Increased methylated *SDC2* (m*SDC2*) has been observed in CRC, with significant differences in measurable amounts of tumor- and serum-derived m*SDC2*, compared to normal adjacent tissue and healthy control samples (32). Subsequent studies have further evaluated the utility of m*SDC2* detection in stool and blood, with a pooled sensitivity and specificity of 0.81 (95%CI: 0.74-0.86) and 0.95 (95%CI: 0.93-0.96), respectively, with sensitivity and specificity remaining consistent across CRC stage (33). Improved detection accuracy of m*SDC2* was observed when used in combination with m*SEPT9.* ColoDefense, a stool-based detection assay using m*SDC2* and m*SEPT9*, has shown a positive detection rate of 90.2-90.9 (area under the curve (AUC), 0.98; 95%CI: 0.95-1.00) (34). However, there is limited information on the utility of measuring both m*SDC2* and m*SEPT9* in blood and, based on the considerable performance in stool, may be worthy targets. Interestingly, a recent study showed high sensitivity (97.3%) for the detection of advanced precancerous lesions and CRC when combining stool-derived m*SDC2*, FIT, and serum-derived CEA (35).

### ALX homeobox 4

*ALX4* is a homeodomain transcription factor typically expressed in the mesenchyme of developing tissues and bones. It has also been found to act as a tumor suppressor, inhibiting proliferation and migration (36). An initial study detected *ALX4* hypermethylation (m*ALX4*) in cfDNA from majority of the polyp and CRC samples, compared to healthy controls, and individuals with hypermethylated tumors were more likely to also have hypermethylated metastases (36). A subsequent study measured *ALX4* methylation in low-volume serum and found 88% sensitivity and 68% specificity for CRC detection, compared to healthy controls. An additional exploratory study evaluating efficacy for polyp and CRC detection found plasma-derived m*ALX4* and m*SEPT9* positivity in 51% and 60% of polyps and CRCs, respectively, compared to 18% in healthy control plasma, using the 2/3 algorithm (37). Of the explored liquid biopsy-based biomarkers, m*ALX4* has demonstrated the greatest efficacy in detection of polyps and preneoplastic lesions.

### Long interspersed nucleotide element-1

*LINE-1* is a retrotransposon that comprises approximately 17% of the human genome. Repression, such as through methylation, allows for active transposable elements and is associated with increased genomic instability (38). In contrast to the previously discussed markers, *LINE-1* hypomethylation has been associated with earlier onset CRC and increased *LINE-1* methylation is associated with improved survival (39). Higher detection of unmethylated *LINE-1* in CRC cfDNA has also been associated with larger tumors, advanced stage and metastasis (40). In support of *LINE-1* hypomethylation as an early detection marker, prior studies have demonstrated a stepwise reduction in *LINE-1* methylation in the transition from normal colonic epithelium to polyp and CRC (41).

### Methylation panels

Individually, liquid biopsy-based methylation markers have low to moderate efficacy for CRC detection; studies utilizing methylation panels have demonstrated improved performance detection. For example, a multicenter cohort study evaluating plasma-derived ctDNA methylation haplotype patterns (20-75 base pair fragments and three or more CpG islands) using a 239 marker panel (ColonES Assay) had a sensitivity of 79% (95% CI: 66.5-87.9%) and 86.6% (95% CI: 80.1-90.9%) for detection of advanced adenoma and CRC, respectively, outperforming carcinoembryonic antigen (CEA) and FIT for all CRC stages (42). Methylations panels, however, do not have to be outrageously extensive. An analysis of The Cancer Genome Atlas (TCGA) CRC dataset identified 13 clustered markers with differential methylation across 11 genes, compared to normal tissue (43). Reducing the panel to eight markers achieved a detection sensitivity and specificity of 95% (95% CI: 76.4-99.7%) and 100% (95% CI: 83.9-100%), respectively, comparing plasma from stage IV CRC to health controls. An additional *in silico* analysis of TCGA data found optimal CRC detection performance using methylation of two nervous system-related genes (*GDNF, SNAP91*) and m*NDRG4*, in conjunction with FIT (44). Finally, a recent cohort study in Brazil demonstrated low but detectable expression of m*SEPT9* and m*BMP3* in CRC across all stages, with an AUC of 0.77, though performance improved when the analysis was restricted to those age 60 and older (45).

Recent advances in methylation-based methods have demonstrated robust improvements in CRC detection specificity and sensitivity. For example, utilization of methylation-specific quantitative PCR (mqPCR) - which detects methylation patterns in CpG islands, in this case 10 regions in the *SEPT9* promotor - showed high detection rates for all stage CRC in an initial technical cohort, but showed slightly lower detection rates in the validation cohort, though this method also demonstrated some efficacy in monitoring recurrence (46). Moreover, methylation-sensitive restriction enzyme digestion followed by sequencing (MRE-Seq), which uses SacII restriction sites to cut unmethylated CpG island recognition sites (CCGCGG), produced 78.1% sensitivity for detecting CRC (AUC 0.978), with stage I-IV sensitivities ranging from 76.2-83.3% (47). An advantage of this technique is a complete evaluation of the global hypomethylation landscape, and most of the markers identified in this screening were located in intron, promoter and intergenic regions (47). Lastly, a combinatorial approach using a 44-marker MRE-Seq signature and validating with mqPCR demonstrated 82% sensitivity and 75% specificity (AUC 0.73) (48). Advanced adenomas could be detected with an adjusted 35-marker methylation signature (AUC 0.80) and a three gene signature could be used to monitor chemotherapy response (AUC 0.90) (48).

Thus, liquid biopsy-derived methylation panels for CRC detection will likely prove more efficacious than individual markers, particularly in determining extent of disease (e.g., certain markers appearing in early-stage CRC versus markers only present in late-stage). There is currently significant discrepancy in the markers included across panels, seeding doubt in the ability to replicate proposed methylation panels across populations. This field – liquid biopsy-based methylation markers for cancer detection – is in its’ infancy, with considerable room for discovery and growth.

## What are the limitations to these methods?

Though this perspective has focused on the use of liquid biopsy-based methylation markers for CRC detection, the issues discussed here are common across most cancer biomarker types. A primary limitation is marker abundance – the target biomarker must be abundant enough to detect in circulation, though not appear so late in disease progression that its detection has no benefit for the patient. Tumor cells may not shed biomarkers at a detectable level, using more conventional methods, until the tumor progresses and invades into the lymphatic system and blood stream. Conversely, there are techniques with the ability to detect/quantify low abundance fragments – such as ddPCR and multiplex mqPCR – but those methods are expensive and generally not used in clinical settings. In the development of these early detection tests – methylation-based or otherwise – there is no consensus which biomarkers to include in creating the optimal detection panel. In our evaluation of methylation-based biomarkers, a small number of biomarkers (e.g., m*SEPT9*) are incorporated in multiple CRC detection panels, but there is little overlap. This, then, begs several questions – what markers are best? Which combination of markers provides the highest, and most accurate, detection? As this information is currently unknown, there is a critical need to address these questions.

Another consideration, particularly for CRC, is the limited ability of available techniques to detect pre-neoplastic lesions (adenomas, advanced adenomas). For example, as mentioned above, despite solid mqPCR assay performance for the 10 *SEPT9* promoter subregions for CRC, detection of polyps and advanced adenoma remained quite low (40%, 23%) (46). An additional study showed detection of m*SEPT9* with m*ADHFE1* (alcohol dehydrogenase, iron containing 1) could better discriminate between healthy, adenoma, and CRC, however specificity remained low for these markers (49). This study interestingly highlights not just the overlapping, but the distinct markers and pathways observed low-grade and high-grade adenomas, adding further complexity to pre-neoplastic lesion/CRC early detection. Thus, more traditional screening strategies, such as colonoscopy and CTC, remain superior for uncovering pre-neoplastic lesions (50).

Finally, but perhaps one of the most critical issues regarding the development and implementation of any cancer detection test, is cost. Recently, the Galleri® liquid biopsy-based multi-cancer early detection (MCED) test, which analyzes circulating cfDNA fragments to determine the presence of cancer and tissue of origin for more than fifty cancer types, came to market as a direct-to-consumer product. Large-scale trials using this test are underway (NHS-Galleri, accrual completed; REACH/Galleri-Medicare, recently announced), but results thus far indicate a high false positive rate (51). Despite its potential utility, Galleri® is not currently FDA-approved, not covered by insurance or Medicare, and is priced at $949. For a sizable proportion of the population, this is an unreasonable amount of money to spend out-of-pocket and may further exacerbate inequities and disparities in cancer screening. In contrast, the median out-of-pocket cost for a colonoscopy, with and without polyp removal, is $104 and $46, respectively (52). For individuals without insurance, this or a similar MCED may be more cost effective than colonoscopy, however FOBT/FIT, which has comparable sensitivity and specificity, has a median out-of-pocket expense of $3.04, not accounting for necessary follow-up procedures (53, 54). Galleri® is a singular example of liquid biopsy-based MCEDs, and many more are in development and clinical testing, with the potential for direct-to-consumer marketing.

## Evolving strategies and conclusions

The use of liquid biopsy for cancer detection is a rapidly evolving field. There is a constant stream of newly identified biomarkers and platforms, however we have yet to optimize those that have already been identified and developed. Methylation biomarkers have shown promise for CRC detection, however a potential strategy to improve detection accuracy is through a multi-omic approach. Blood samples contain an array of measurable biomarkers – cfDNA, extracellular vesicles/exosomes, secreted proteins, lipids, metabolites, epigenetics, immune cells, microbes – all of which may be informative to CRC detection. An increasing number of studies continue to show differential expression of multi-omic biomarkers in CRC cases, compared to normal controls (55). A limitation to a multi-omic approach may be that incorporating numerous biomarker types into a singular platform may be methodologically difficult, though progress is being made using microfluidic systems. In summary, advancements in CRC screening may come with blood-based biomarkers, though substantial research, both at the bench and at a population scale, is needed to improve the existing tools.

## Data availability statement

The original contributions presented in the study are included in the article/supplementary material. Further inquiries can be directed to the corresponding author.

## Author contributions

HL-K: Conceptualization, Writing – original draft.
